# Antiseptic Surgery

**Published:** 1890-01

**Authors:** W. F. Westmoreland

**Affiliations:** Lecturer on Minor Surgery, Atlanta Medical College


### Southern Medical Record.

A MONTHLY JOURNAL OF MEDICINE AND SURGERY.

VOL. XX. ATLANTA, GA., JANUARY, 1890. No. 1.

### Original and Selected Articles.

### ANTISEPTIC SURGERY.

BY W. F. WESTMORELAND, JR., LECTURER ON MINOR SURGERY, ATLANTA MEDICAL COLLEGE.

The object which we will endeavor to keep steadily in view in this paper is that it shall be essentially practical; we shall endeavor to present this subject to you as clearly as possible. In doing this, in order to keep our subject within reasonable limits, we will be compelled to be, to a certain extent, dogmatic; but we will lay down those rules for your guidance, which we have found from our own clinical experience to be most efficient. If this paper shall enable you to obtain a clear and comprehensive knowledge of this subject, which shall prove a trustworthy guide to you in your surgical work, the ends which we have in view will be accomplished, and we shall feel amply rewarded for our labors.

We will not enter into a discussion of the theory of antiseptics, or of its merits. Its value stands unchallenged among intelligent surgeons. Germany not only recognizes it, but compels its use; there it is a misdemeanor, punishable by a fine, for a surgeon to operate without using antiseptic precautions.

I read a paper on this subject several years ago, and it affords me the greatest pleasure to see surgeons who were then its bitterest opponents, now class themselves amongst its warmest advocates.

Do not fall into the error of thinking that antiseptic surgery only involves thorough cleanliness and disinfection of patient, instruments, surroundings, etc. There are other points which are just as important.

Antiseptic surgery is a system that involves the consideration of THREE ESSENTIAL POINTS.

I. Thorough antisepsis, not only of the field of operation, instruments and wound, but carried out during every detail of the operation, and at subsequent dressings.

II. Thorough drainage, for the complete removal of all the secretions from the wound.

III. A dressing that will insure the absorption of all secretions, compression of the wound and complete immobilation of the surrounding parts.

The non-observance of either of these will defeat the object after which we are striving, viz.: Primary union throughout the wound, without inflammation or suppuration.

I have found numbers of physicians who had the erroneous impression that good antiseptic work can not be accomplished outside of hospitals, or at least cannot be carried out by the surgeon who has to operate first here then there, in a city today and some hovel in the country to-morrow. That the paraphernalia necessary is so much and so cumbersome that it can't be carried from place to place, and that the cost of dressing is such that only the rich can afford to buy them.

There is no hovel so poor, but can supply a few tin pans or platters in which to place the instruments, bowls for sponges and towels, and a convenient well with a bountiful supply of water with which to make solutions. So the "cumbersome paraphernalia" is lessened to a few bottles of solutions, instruments, sponges and dressings, which are conveniently carried in a hand satchel.

As to the great expense, we can mention this experience, when we wished to introduce antiseptics into clinic, it was objected to on account of costing too much; we did not think there would be much difference. In comparing the expense of the clinic at the end of the year, it was found that the cost lacked a fourth of being as great as when the old method was used; although there had been a larger number of operations and many of them of a more serious character.

But surely the lessened amount of pain, the shortness of the confinement to bed, the greater rapidity of union of the wound, and the quickness with which the patient is returned to work, should be compensations that would outweigh any idea of trouble or cost.

Before going further, we will for the sake of clearness and conciseness, sub-divide our paper into two parts.

I. ANTISEPTIC TREATMENT OF WOUNDS; PREPARATION OF ANTISEPTIC MATERIAL.

2. Antiseptic treatment of wounds. The manner in which we have taken up these divisions, viz.: the treatment of wounds before the preparation of materials, has somewhat the appearance of "hitching the cart before the horse." But we think after seeing the effect, one is more prone to closely study the cause.

We will divide wounds into two general classes as regards their nature; each will at first have to be separately considered:

Accidental Wounds;

Wounds Made During Operations.

And again divide them as regards their treatment, into

Wounds to be closed, in which we expect primary union;

Wounds to be left open, in which we expect to heal by granulation.

ACCIDENTAL WOUNDS. This class of injuries come to your hands already infected, by what ever substance has infected the wound, or the dressings which are put on by some companion, and frequently particles of foreign matter are carried into, and remain in the wound.

In these patients, after an examination, if it is considered necessary, they are etherized, and while this is being done, the preparatory steps for cleaning the wound and arranging instruments can be carried out.

When ready, the wound is thoroughly cleansed of all blood, clots, and particles of foreign matter, &c. If the external opening is not sufficiently large to allow the deepest recesses of the wound to be easily reached and thoroughly cleaned, *cut it larger* until it is. There is absolutely no excuse, in trying to clean a large, deep seated wound through a small external opening, nor will any effort to prevent suppuration in wounds treated this way prove satisfactory. After cleansing the wound with the weak solution, and stopping hemorrhage, wash it out with bichloride sol. (1-500), using only enough to reach into all the recesses, and immediately rewash it with the weaker solution, (1-2000), using fingers to gently rub the surfaces of the wound, to thoroughly remove the stronger solution, which will cause poisoning, if left to be absorbed. I may say here, that throughout this paper, when the word *solution* is used *bichloride mer. sol.* is *meant*, unless otherwise specified, the numeral afterwards indicating the strength.

The wound is now as clean and free from infection as if it had been made with thorough antiseptic surroundings. These wounds are most frequently contused, or lacerated, or sometimes both varieties are combined. When it is possible I pare the edges, trim out the contused or lacerated tissue, and change its character to that of an incised wound as much as possible, so as to bring the edges, which will now have sufficient vitality for rapid union, into direct coaptation and treat as closed wounds. In punctured wounds; and some varieties of contused, where they are so badly crushed that they can not be trimmed without too much loss of tissue, it is generally better to leave them alone and treat as open wounds.

WOUNDS THE RESULT OF OPERATIONS.

In these cases there is time to make all preparations for the operation; as operative wounds are nearly always closed; I will combine under this head wounds to be closed, and operative wounds, and illustrate by describing an amputation of the leg, which involves the wounding of both the soft and hard parts:

We have decided to amputate Mr. Smith's leg at the juncture of the upper and middle third, to-morrow. Before he leaves the office the leg has been shaved, washed with solution (1-1000), scrubbed with a little ether, to remove the oily excretions from the pores, rewashed with the same solution and applied a light antiseptic dressing, so as to have leg in good condition for operation. Also tell him to have plenty of hot water, a few bowls and platters well washed and two large pitchers in the room at appointed time. In preparing for operation, besides necessary instruments, we take a bottle of carbolic acid, half dozen prepared sponges; a bottle containing two sizes of cat gut, Nos. 1 and 2; two or three large rubber drainage tubes; a can of bichloride gauze, couple of pounds of borated cotton; a dozen bichloride bandages 3 inches wide, and half a dozen starch bandages same width, a fountain syringe, and two rubber cloths. When we reach Mr. Smith's house, I see if they have a table large enough to use as an operating table, if not can use the bed, find they have a kitchen table which suits very well. Place a quilt or blanket on it, over which place one of the rubber cloths; the table is ready, and while the assistants are putting the patient on it and etherizing him, arrange instruments, solution, &c. Arrange instruments in the platters and cover them with carbolic acid solution (1-20) the last thing before beginning the operation, add just as much more water, which weakens solution (1-40). Make up bichloride mer. solution next. Secure a large pitcher holding two or three quarts of water; if you know its capacity it will save measuring each time you wish to make solution. The bichloride mer. tablets each contain seven and a half grains of mercury. One of them to a quart of water gives one to two thousand solution.

Fill your pitcher with hot water and dissolve in it one tablet for each quart of water, this will give you solution (1-2000). I never use a stronger solution in operating. Fill the fountain syringe with this solution and hang it in convenient reach, about three feet above the field of operation. This gives fall enough.

Fill four bowls with solution; into one, put Esmarch's bandage and retractor, put your sponges in another, three or four towels in the third, and keep the other to wash hands in from time to time during the operation to keep them free from blood.

Arrange a rubber cloth around the limb, carrying it high up under the leg, so that it will carry the blood and water into a tub at the foot of table.

It is best to have three assistants besides the ether giver, one to hold limb, another to hand instruments, and the third to wash sponges and douche the wound when necessary.

The last thing before beginning operation, see that your own and assistant hands and nails are thoroughly scrubbed and cleansed with soap and nail-brush—be especially particular about cleansing nails, for it is surprising how much *infecting material* can be carried under a *finger-nail,* and wash well in solution (1-2000). Assistants in position, instruments and catgut on small table in easy reach. Take off light dressing and wash leg with solution (1-2000). Wrap towel taken out of bowl around foot and leg, and apply Esmarck's bandage, carrying it up above knee and securing it. It is a good idea to have leg elevated while applying bandage, as gravity will assist it in forcing the blood out of the limb. After securing it at the point mentioned, remove all the bandage below and place a few towels around field of operations, so that instruments and hands will only touch antiseptic material. Give the leg a final douching from fountain syringe before beginning. We make circular flap operation, with lateral incision preferably on the outer side; this incision enables you to more easily retract the flap. We have found no advantage in making a periosteal flap, and it unnecessarily delays the operation. During the operation we douche the field of operation as often as necessary to keep it free from blood. After the section of the bone, we catch up and ligate all the arteries possible, before removing the Esmarck's bandage, ligating the principal vessels with the larger size catgut (No. 2), and the arterioles with the smaller catgut (No. 1). If there is much venous hemorrhage, I also ligate the veins. If, after removing Esmarck's bandage, there is any continuation of the hemorrhage, I continue ligating every bleeding point. Tie catgut ligature with three knots instead of two—it will prevent slipping—and cut them off short.

Thoroughly douche the wound; the effect of the solution is to stop the oozing which always occurs after using Esmarck's bandage, and will give you a dry, glazed surface. If oozing continue, I stuff the flaps with sponges wrung out of solution (1-2000) as hot as the wound will stand, and leave them in for a few minutes. This generally stops it entirely.

I now sew up the flap, first with a large strand of catgut (No. 2), taking deep, continuous sutures, making the insertions of the needle about a half or three-quarters of an inch apart. Then, with a smaller strand (No. 1), I take a superficial continuous suture, just going through the skin, and close enough together to bring the edge of the flap into direct coaptation.

I put two large rubber drainage tubes—one long and the other short. This insures drainage of all parts of the wound through the lateral incision. Hold them in position by passing a safety-pin through them; this will keep them from slipping into the wound, but will not prevent them coming entirely out. I think the better plan is to hold them in position by stitching them to the edge of the wound with catgut, which holds them firmly.

Now wash out by putting the nozzle of the syringe in the opening of the longer tube; if the solution come out of the shorter one freely, you can feel satisfied as to the perfect drainage of the wound; if it does not come out freely, change position of the tube until it does.

Now we are ready for the dressing. The bichloride gauze comes about eight layers thick; cut four pieces a little wider than the breadth of the leg and long enough to extend six or eight inches up the leg above the stump. The first piece put across the stump laterally, so as to extend up either side of the leg; then a piece crossing that, so as to extend up ante-posteriorly; firmly bandage with a bichloride bandage; arrange the other two pieces in the same manner, and again bandage firmly, using two or three bandages if necessary. Now put on two or three layers of borated cotton in the same manner, only cutting the pieces much longer, so that they will reach up about the middle third of the thigh. By having them this long it will prevent the tendency of the dressings to slip over the end of the stump; bandage each successive layer of the cotton on firmly with a bichloride bandage. I now put the limb on a well padded posterior straight splint extending from the middle third of the thigh to a few inches beyond the stump. This prevents any contraction of the knee, which is sometimes very troublesome, causing the leg to rest on the stump; keeps the limb perfectly quiet, so there is no muscular spasm; the projecting splint prevents the stump being struck, and adds very much to the comfort of the patient, as the limb can be lifted about, when he desires to change position, without any pain. Unless there are indications to the contrary, the dressing is not disturbed until the seventh or eighth day.

The indications for a change are a continued elevation of pulse or temperature after the second day, or a sudden rise of either after this time, that cannot be accounted for satisfactorily in any other way than by some disturbance in the wound. We always have a reactionary fever after any important operation, but it is generally easily controlled by a couple of drops of Norwood's tinct. veratrum vir., during the first thirty-six or forty-eight hours. If it persists longer than this, remove the dressing and examine the wound. When this is necessary, redress in the same manner previously mentioned.

About the seventh or eighth day, I take off the dressing, wash out the wound gently. If you use a strong current of water, you will distend the flap and break up the partial union which has already taken place. Remove the drainage tubes, and redress in the same manner as at first, using every antiseptic precaution while it is being done. This dressing is only necessary to remove the drainage tubes. When bone drainage tubes, which are absorbable, can be used, there is no necessity of removing dressing until the wound is well.

The second dressing remains on for a week or ten days longer, and when removed at that time, the wound is generally found well. If not completely well, however, a lighter dressing can be used.

The indications for dressing any other wounds are the same. Thorough drainage through rubber or bone tubes, several layers of gauze, over which are placed the layers of cotton, each successive layer being firmly bandaged on with bichloride bandages; and when dressings are removed, redressing them in the same manner.

Wounds dressed in this manner always give, as the result, quick primary union under one or two dressings. This is a rule to which there will be but few exceptions; and in my hands, when there is an exception, I always blame myself for the neglect of some antiseptic precaution, and not the dressings.

WOUNDS WHICH ARE TO BE LEFT OPEN.

Unfortunately, we sometimes have wounds to treat where, through the necessary loss of tissue, there is not flap enough to cover the wound, or other reasons, as in badly contused wounds, where the tension necessary to draw the edges together would cause their necrosis.

When such wounds are the results of an accident, the steps for preparatory cleansing are the same as already mentioned, and the wound dressed in the same manner as after an operation.

The plan I follow is, after stopping hemorrhage and washing with solution (1-2000), to dust a thin layer of iodoform over the whole denuded surface, then pack it gently with loose pieces of iodoform gauze, seeing that all the crevices are filled, packing it even with the surrounding surfaces. Then the same dressing of bichloride gauze and borated cotton is applied and firmly bandaged in the same manner already mentioned. The dressings can stay on the same length of time; but, of course, as the wound has to heal by granulation, it takes much longer to get well.

All the dressings should extend eight or ten inches from the edges of the wound, otherwise the secretion from it will ooze through the edges of the dressing and necessitate its early removal.

*To be continued.*

